# Supplementary material for: Pasture vs. Coop: Biomarker Insights into Free-Range and Conventional Broilers
Source: Animals (Basel). 2024 Oct 24;14(21):3070. doi: 10.3390/ani14213070 (PMC11544995; doi:10.3390/ani14213070)
Supplement: Supplementary file 1 [file animals-14-03070-s001.zip › Table S3.pdf]

Regression type: **Logistic regression**

Model				
Parameter estimates	Variable	Estimate	Standard error	95% CI (profile likelihood)
β0	Intercept	1.166	0.4898	0.2287 to 2.161
β1	**CPK**	-0.7977	0.1062	-1.027 to -0.6082
β2	**LPS**	2.195	0.3828	1.497 to 3.007
Odds ratios	Variable	Estimate	95% CI (profile likelihood)	
β0	Intercept	3.209	1.257 to 8.682	
β1	CPK	0.4504	0.3581 to 0.5443	
β2	LPS	8.98	4.467 to 20.22	

Model diagnostics	Degrees of Freedom	AICc		
Intercept-only model	299	417.9		
Selected model	297	165.8		

Area under the ROC curve	
Area	**0.9547**
Std. Error	0.01021
95% confidence interval	0.9347 to 0.9747
P value	<0.0001
Negative predictive power (%)	**89.86**
Positive predictive power (%)	**83.95**

Data summary	
Rows in table	300
Rows skipped (missing data)	0
Rows analyzed (#observations)	300
Number of FR	150
Number of C	150
Number of parameter estimates	3

Hypothesis tests	Statistic	P value	Null hypothesis	Reject Null Hypothesis?
Hosmer-Lemeshow	2.461	0.9635	Selected model is correct	**No**

Table S3: Results of Multiple logistic regression analysis for the combinatorial use of IgM anti-LPS NAbs and CPK levels.

In all cases, the significance level was set at 5%, the tests were two sided and a result was considered significant if the estimated p-value was less than the significance level ($p<0.05$). Statistical analysis was performed and graphs were made in GraphPad Prism version 9.0.0 (GraphPad Software, San Diego, California USA).
