# Supplementary material for: Pasture vs. Coop: Biomarker Insights into Free-Range and Conventional Broilers
Source: Animals (Basel). 2024 Oct 24;14(21):3070. doi: 10.3390/ani14213070 (PMC11544995; doi:10.3390/ani14213070)
Supplement: Supplementary file 1 [file animals-14-03070-s001.zip › Figure S1.pdf]

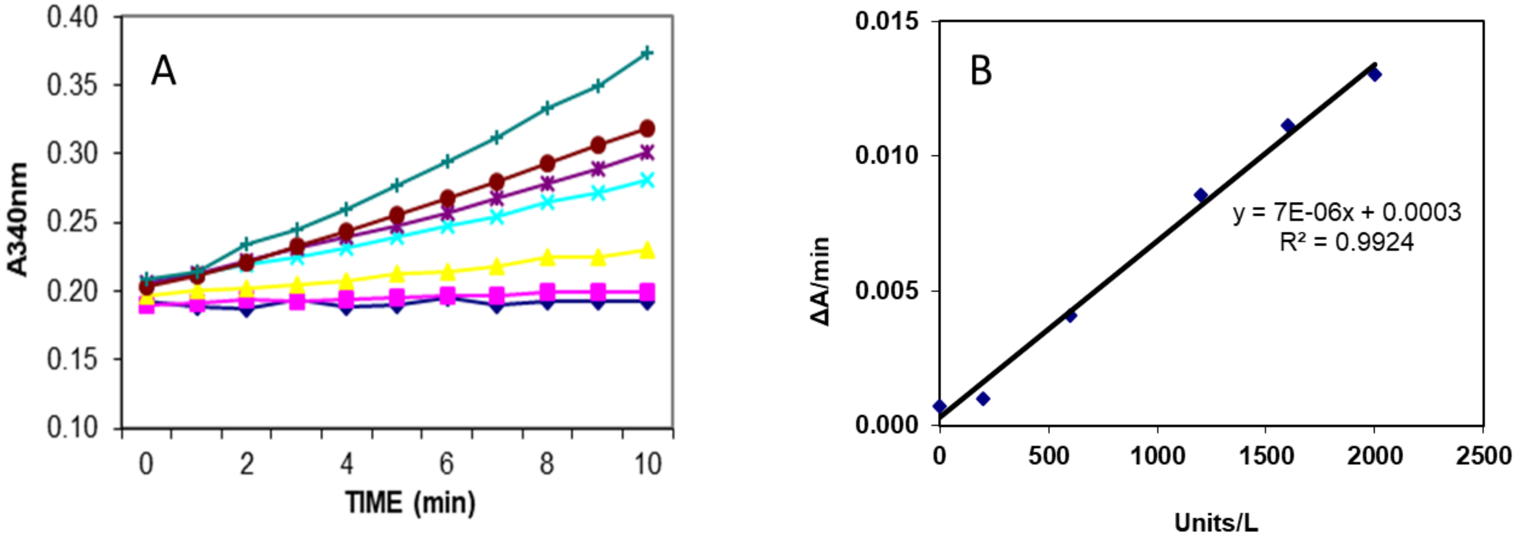

Figure S1: Representative standard curve of CPK enzyme activity. a. NADPH formation per time of increased standards CPK of known activity. b. Linear standard curve (y=ax+b) is constructed by ΔA/min per enzyme activity of the standards. One CPK enzyme unit (1 Unit) is the amount of enzyme that will transfer 1.0 mmole of P from phosphocreatine to ADP per minute at pH 6.0. Linear range 30-1,800 Unit / L.
