# Supplementary material for: Pasture vs. Coop: Biomarker Insights into Free-Range and Conventional Broilers
Source: Animals (Basel). 2024 Oct 24;14(21):3070. doi: 10.3390/ani14213070 (PMC11544995; doi:10.3390/ani14213070)
Supplement: Supplementary file 1 [file animals-14-03070-s001.zip › Table S1.pdf]

Regression type: **Logistic regression**

Model				
Parameter estimates	Variable	Estimate	Standard error	95% CI (profile likelihood)
β0	Intercept	-2.316	0.3157	-2.965 to -1.723
β1	**LPS**	1.927	0.2495	1.465 to 2.446
Odds ratios	Variable	Estimate	95% CI (profile likelihood)	
β0	Intercept	0.09863	0.05158 to 0.1785	
β1	LPS	6.871	4.326 to 11.54	

Model diagnostics	Degrees of Freedom	AICc		
Intercept-only model	299	417.9		
Selected model	298	325.9		

Area under the ROC curve	
Area	**0.8292**
Std. Error	0.02531
95% confidence interval	0.7796 to 0.8789
P value	<0.0001
Negative predictive power (%)	**79.75**
Positive predictive power (%)	**83.1**

Data summary	
Rows in table	300
Rows skipped (missing data)	0
Rows analyzed (#observations)	300
Number of FR	150
Number of C	150
Number of parameter estimates	2

Hypothesis tests	Statistic	P value	Null hypothesis	Reject Null Hypothesis?
Hosmer-Lemeshow	57.84	<0.0001	Selected model is correct	**Yes**

Table S1: Results of Multiple logistic regression analysis for IgM anti-LPS NAb levels;

In all cases, the significance level was set at 5%, the tests were two sided and a result was considered significant if the estimated p-value was less than the significance level ($p<0.05$). Statistical analysis was performed and graphs were made in GraphPad Prism version 9.0.0 (GraphPad Software, San Diego, California USA).
