# Supplementary material for: Pasture vs. Coop: Biomarker Insights into Free-Range and Conventional Broilers
Source: Animals (Basel). 2024 Oct 24;14(21):3070. doi: 10.3390/ani14213070 (PMC11544995; doi:10.3390/ani14213070)
Supplement: Supplementary file 1 [file animals-14-03070-s001.zip › Table S2.pdf]

Regression type: **Logistic regression**

Model				
Parameter estimates	Variable	Estimate	Standard error	95% CI (profile likelihood)
β0	Intercept	3.449	0.3943	2.724 to 4.277
β1	**CPK**	-0.6579	0.08089	-0.8312 to -0.5125
Odds ratios	Variable	Estimate	95% CI (profile likelihood)	
β0	Intercept	31.46	15.25 to 72.05	
β1	CPK	0.518	0.4355 to 0.5990	

Model diagnostics	Degrees of Freedom	AICc		
Intercept-only model	299	417.9		
Selected model	298	214.6		

Area under the ROC curve	
Area	**0.8966**
Std. Error	0.01936
95% confidence interval	0.8587 to 0.9346
P value	<0.0001
Negative predictive power (%)	**94.49**
Positive predictive power (%)	**82.66**

Data summary	
Rows in table	300
Rows skipped (missing data)	0
Rows analyzed (#observations)	300
Number of FR	150
Number of C	150
Number of parameter estimates	2

Hypothesis tests	Statistic	P value	Null hypothesis	Reject Null Hypothesis?
Hosmer-Lemeshow	24.52	0.0019	Selected model is correct	**Yes**

Table S2: Results of Multiple logistic regression analysis for CPK levels;

In all cases, the significance level was set at 5%, the tests were two sided and a result was considered significant if the estimated p-value was less than the significance level ($p<0.05$). Statistical analysis was performed and graphs were made in GraphPad Prism version 9.0.0 (GraphPad Software, San Diego, California USA).
